# Jackstone: A Calculus “Toy” in the Bladder. A Case Report of Rare Entity and Comprehensive Review of the Literature

**DOI:** 10.15388/Amed.2021.29.1.6

**Published:** 2022-01-26

**Authors:** Evangelos N. Symeonidis, Dimitrios Memmos, Anastasios Anastasiadis, Ioannis Mykoniatis, Eliophotos Savvides, Georgios Langas, Panagiotis Baniotis, Athanasios Bouchalakis, Stavros Tsiakaras, Panagiotis Stefanidis, Michail Stratis, Wilbert F. Mutomba, Ioannis Vakalopoulos, Georgios Dimitriadis

**Affiliations:** 1st Department of Urology, Aristotle University of Thessaloniki, School of Medicine, “G. Gennimatas” General Hospital, Thessaloniki, Greece; 1st Department of Urology, Aristotle University of Thessaloniki, School of Medicine, “G. Gennimatas” General Hospital, Thessaloniki, Greece; 1st Department of Urology, Aristotle University of Thessaloniki, School of Medicine, “G. Gennimatas” General Hospital, Thessaloniki, Greece; 1st Department of Urology, Aristotle University of Thessaloniki, School of Medicine, “G. Gennimatas” General Hospital, Thessaloniki, Greece; 1st Department of Urology, Aristotle University of Thessaloniki, School of Medicine, “G. Gennimatas” General Hospital, Thessaloniki, Greece; 1st Department of Urology, Aristotle University of Thessaloniki, School of Medicine, “G. Gennimatas” General Hospital, Thessaloniki, Greece; 1st Department of Urology, Aristotle University of Thessaloniki, School of Medicine, “G. Gennimatas” General Hospital, Thessaloniki, Greece; 1st Department of Urology, Aristotle University of Thessaloniki, School of Medicine, “G. Gennimatas” General Hospital, Thessaloniki, Greece; 1st Department of Urology, Aristotle University of Thessaloniki, School of Medicine, “G. Gennimatas” General Hospital, Thessaloniki, Greece; 1st Department of Urology, Aristotle University of Thessaloniki, School of Medicine, “G. Gennimatas” General Hospital, Thessaloniki, Greece; 1st Department of Urology, Aristotle University of Thessaloniki, School of Medicine, “G. Gennimatas” General Hospital, Thessaloniki, Greece; 1st Department of Urology, Aristotle University of Thessaloniki, School of Medicine, “G. Gennimatas” General Hospital, Thessaloniki, Greece; 1st Department of Urology, Aristotle University of Thessaloniki, School of Medicine, “G. Gennimatas” General Hospital, Thessaloniki, Greece; 1st Department of Urology, Aristotle University of Thessaloniki, School of Medicine, “G. Gennimatas” General Hospital, Thessaloniki, Greece

**Keywords:** Jackstone, Vesical Calculus, Bladder Calculus, Bladder stone disease, Benign Prostate Hyperplasia, Prostatectomy, „Jackstone“, šlapimo pūslės akmuo, akmenligė, gerybinė prostatos hiperplazija, prostatektomija

## Abstract

**Background:**

An uncommon type of urinary calculus, Jackstone was named after its distinct resemblance to the children’s game “Jacks.” It typically involves the bladder and, to a lesser extent, the upper urinary tract.

**Case Presentation:**

Herein, we report a case of Jackstone vesical calculus in a 75-year-old male undergoing elective open prostate surgery for benign prostate hyperplasia refractory to medical treatment. Preoperative clinical examination revealed intermittent gross hematuria and symptoms suggestive of bladder outlet obstruction, while radiological investigation confirmed the presence of a solitary star-shaped spike-like bladder stone along with an overly enlarged prostate. Following open simple prostatectomy and concomitant intact stone removal, our patient made an uneventful postoperative recovery.

**Conclusion:**

This case highlights an infrequent subtype of bladder lithiasis and further expands upon the importance of promptly treating the underlying cause once this rare entity is detected. A comprehensive review of the literature on Jackstone calculi is further presented.

## Santrauka.

**Problema.:**

Neįprastas šlapimo pūslės akmuo buvo pavadintas „Jackstone“, nes priminė vaikų žaislą „Jacks“. Dažniausiai jis randamas šlapimo pūslėje, o rečiau – viršutiniuose šlapimo takuose.

**Atvejo pristatymas.:**

Šiame straipsnyje pranešame apie „Jackstone“ šlapimo pūslės akmenį, aptiktą 75 metų vyrui, kuriam atlikta pasirinktinė atvira prostatos operacija, nustačius gerybinę prostatos hiperplaziją, kuri nepasidavė gydymui medikamentais. Prieš operaciją atlikus klinikinį tyrimą, nustatyta protarpinė intensyvi hematurija, o simptomai leido įtarti šlapimo pūslės išėjimo obstrukciją. Radiologinis tyrimas patvirtino vieno žvaigždės formos, spygliuoto šlapimo pūslės akmens ir padidėjusios prostatos diagnozę. Atlikus paprastą prostatektomiją ir kartu pašalinus visą akmenį, paciento pooperacinė sveikimo eiga buvo sklandi.

**Išvada.:**

Šis atvejis pabrėžia nedažną šlapimo pūslės akmenligės potipį ir plačiau paaiškina, kaip svarbu greitai taikyti tinkamą gydymą, kai šis retas atvejis yra nustatomas. Toliau pateikiama išsami literatūros apžvalga apie „Jackstone“ akmenis.

## Introduction

The complex pathophysiological mechanisms involved in urinary stone formation never cease to amaze urologists. Despite being previously described, Jackstone calculi remain a rare and underreported form of urolithiasis [1]. The term “Jackstone” was aptly coined to illustrate their similarity with the children’s toy “Jacks” [2,3]. Since the first recorded case by Everidge, back in 1927, a rising number of studies shed light on this uncommon type of urinary calculus [4]. Herein, we present a Jackstone bladder calculus retrieved from a 75-year-old male undergoing elective open simple prostatectomy due to benign prostate hyperplasia (BPH) refractory to medical treatment. In addition, we discuss the diagnostic and management considerations of similar cases cited in the world literature. This case report has been reported in line with the SCARE criteria [5].

## Case Presentation

A 75-year-old Caucasian male attending the outpatient prostate clinic at our hospital underwent an elective prostate surgery due to BPH refractory to combination medical treatment. He reported a history of transient episodes of painless gross hematuria that were self-limited. He also complained of obstructive lower urinary tract symptoms (LUTS) aggravation during the last ten months.

**Fig. 1.**
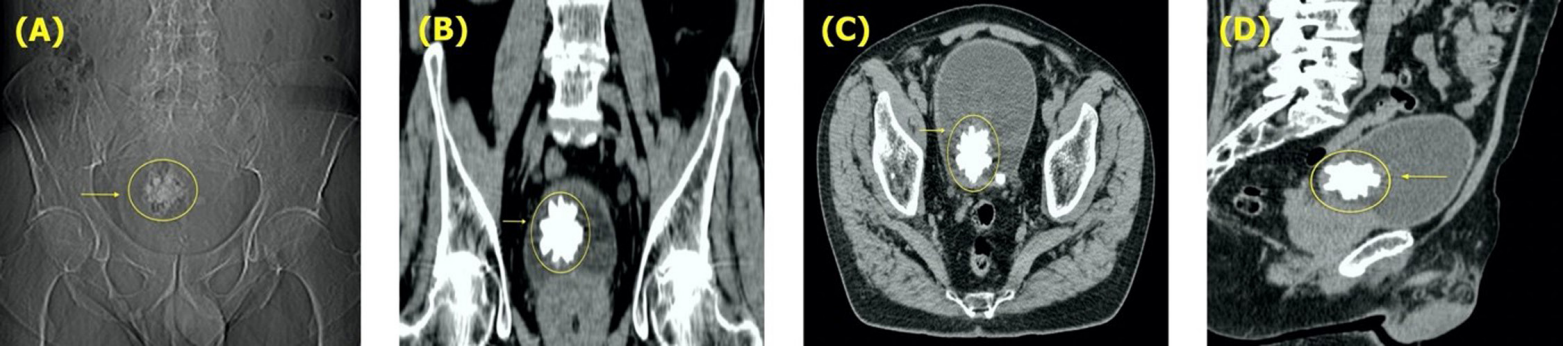
(**A**) Plain X-ray (KUB) demonstrating radiopaque pelvic shadow with sharp and spiculated margins (yellow arrow, and circle). Computed tomographic (CT) images. (**B**) Coronal, (**C**) Axial, and (**D**) Sagittal planes depicting the solitary star-shaped, spiculated Jackstone inside the bladder and intravesical protrusion of the prostate gland (yellow arrow, and circle).

**Fig. 2.**
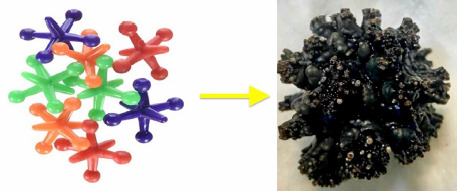
Postoperative image demonstrating the retrieved Jackstone. Note its resemblance to the popular children’s game “Jacks”.

He had no previous significant surgical history and denied any history of smoking. No remarkable medical or urological history was present at that time. Due to LUTS investigation months before the scheduled surgery, ultrasonography, computed tomography (CT), and cystoscopy were performed. As shown in *Fig.1*, the CT scan revealed a solitary star-shaped, spiculated Jackstone inside the bladder with no other pathological findings aside from an overly enlarged prostate (130 ml).

We completed the standard preoperative workup, and laboratory examination was within normal limits. In addition, kidney-ureters-bladder (KUB) radiography identified the already confirmed CT finding demonstrating a single radiopaque pelvic shadow with sharp and spiculated margins.

After obtaining informed consent and thoroughly analyzing the various treatment options based on the stone and prostate size, the patient elected to proceed with an open transvesical prostatectomy. A single 3.5×3.5×3 cm bladder calculus was concurrently removed with macroscopic features suggestive of the Jackstone calculus (JC) *(Fig.2)*. Notably, the stone was black and unusually light, exhibiting a mamillated appearance.

There were no immediate perior postoperative complications, and the patient was finally discharged home on postoperative day 7. Apart from the uneventful recovery, no other abnormality was detected during the 6-month follow-up. More specifically, LUTS showed complete remission, while the quality of life significantly improved.

## Discussion

Urolithiasis constitutes a major health problem with an estimated prevalence between 1 and 20% globally [6]. Bladder stones account for 5% of urinary calculi and can be generally classified into three distinct groups; migratory, primary, and secondary [7]. They can be either single or multiple and usually vary in size, shape, and color. The clinical presentations include nonspecific abdominal pain, voiding and obstructive LUTS, suprapubic pain, and episodes of macroscopic hematuria [7,8]. In some instances, bladder calculi can manifest without symptoms [8,9].

The pathogenesis is considered multifactorial, with bladder outlet obstruction secondary to benign prostate hyperplasia being a plausible mechanism for stone formation in the vast majority of cases [7,10]. Outflow hindrance leads to incomplete bladder emptying, urine stagnation, and urinary debris accumulation, with the organic matter subsequently acting as a nidus for crystal formation [7]. Other recognized predisposing factors are neurogenic bladder dysfunction, recurrent urinary tract infections, chronic catheterization, urethral strictures, and intravesical foreign bodies [1,10].

Jackstone calculi comprise an infrequent form of vesical lithiasis, morphologically characterized by an atypical shape of elongated protuberances resembling children’s “Jacks” toy *(Fig.1)* [1,11]. Their mamillated appearance has led others to delineate JC as mulberry stones [12]. Although commonly described in the veterinary literature, JC remain an exceptionally under-reported entity in humans [8,13]. Furthermore, the sheer protrusions (jack arms), comparable to spines, may provoke chronic bladder urothelium irritation, inflammation, and edema, further resulting in glycosaminoglycan layer disruption and risk for malignancy [2,7]. Lately, Canela et al. purported to elucidate the nature of JC, regarding their mineral and organic composition. Using nondestructive microcomputed tomography and infrared spectroscopy, the authors were able to propose a molecular basis explaining the bizarre morphology of JC. They have found that each arm had an X-ray lucent core with thin layers of apatite transversely arranged towards the arm’s axis. They also hypothesized that the protein-rich core of the jack spike might preferentially bind more protein from the urine and resist deposition of calcium oxalate, in a way allowing its faster linear growth compared to the body of the stone. Therefore, as more proteins deposit onto the tip of the arm, more calcium oxalate monohydrate layers are laid, driving linear extension outward from the stone’s body [14].

A comprehensive literature review, limited to English-only studies, was performed at the end of August 2021 within the Medline via PubMed database using the keyword search term “jackstone calculus.” No specific time boundaries were applied, while animal studies or studies with an irrelevant topic and insufficient data were excluded from further analysis. Initially, 19 articles were identified, 8 of which were excluded based on our search criteria. The remaining 11 studies included in our review were considered eligible for full-text assessment. Additional articles were retrieved by hand searching from reference lists of the eligible studies. A synopsis of studies reporting on Jackstone bladder calculi is presented in *Table 1*. Overall, patients experiencing this pathology had a mean age of 73 (range: 55–84), and the majority (78.6%) was found to have a single JC. Concurrent bladder stone removal and prostate surgery were the treatment of choice in approximately half of the patients (42.9%), while open cystolithotomy was solely attempted in a roughly equal percentage (35.7%).

Recently, Muneeb et al. reported one of the largest Jackstones to date, measuring 6x6cm, in a 55-year-old male presenting with worsening voiding LUTS. Interestingly, the patient was solely managed with open cystolithotomy and opted for postoperative BPH treatment with tamsulosin [15]. Similarly, Singh et al. presented the retrieval of a JC from a 60-year-old patient treated with open cystolithotomy and subsequent initiation of alpha-blocker therapy [8].

In 2018, Heathcote et al. demonstrated a successful simultaneous transurethral resection of the prostate (TURP) and cystolitholapaxy in a 78-year-old male presenting to the emergency department with a 2-week intermittent gross hematuria, bladder fullness, urgency, and suprapubic tenderness [16]. Likewise, Subasinghe et al. dealt with a 67-year-old patient suffering from deteriorating voiding LUTS and a single episode of painful macroscopic hematuria. They proceeded with concomitant cystolitholapaxy and TURP, underlining an uneventful postoperative recovery [1].

**Table 1.** Studies reporting on Jackstone bladder calculi

Author, Year	Country	Age (years)	Number of stones	Symptoms	Diagnostic Modality	Treatment
Present case, 2021	Greece	75	S	Intermittent gross hematuria, Obstructive LUTS	CT, US, KUB X-ray, Cystoscopy	Open prostatectomy and stone removal
Muneeb et al., 2021 [15]	Pakistan	55	S	Voiding LUTS	US, KUB X-ray	Open cystolithotomy
Banerji et al., 2019 [11]	USA	75	M	3-year history of poor urinary flow, hematuria, urgency	CT, Cystoscopy	Laser cystolithoripsy and concurrent TURP
Carneiro et al., 2019 [17]	Portugal	77	S	Incidental finding	US, CT	Stone removal
Brogna et al., 2018 [9]	Italy	84	S	Nonspecific abdominal pain	CT	Patient refused treatment
Heathcote et al., 2018 [16]	USA	78	S	Intermittent gross hematuria, bladder fullness, urgency, suprapubic tenderness	CT	Cystolitholapaxy and concurrent TURP
Roose et al., 2018 [3]	Belgium	82	S	Gross hematuria	TRUS, CT	Patient refused treatment
Subasinghe et al., 2017 [1]	Sri Lanka	67	S	Worsening voiding LUTS, Gross hematuria	US, KUB X-ray	Cystolitholapaxy and concurrent TURP
Singh et al., 2011 [8]	India	60	S	Gross hematuria, intermittent episodes of urinary retention	US, KUB X-ray	Open cystolithotomy
Wong et al., 2010 [24]	UK	83	S	Gross hematuria	KUB X-ray, Cystoscopy	Open cystolithotomy
Perlmutter et al., 2002 [13]	USA	75	S	Incidental detection	US, KUB X-ray, CT	Urologist referral
Gane et al. 1969 [12]	UK	64	S	Incidental detection	KUB X-ray	Open cystolithotomy
Stewart et al. 1961 [25]	UK	69	M	Urine discoloration	KUB X-ray, Cystoscopy	Open prostatectomy and stone removal
Everidge et al. 1927 [4]	UK	78	M	n.a	n.a	Open prostatectomy and stone removal

S: Single; M: Multiple; CT: Computed Tomography; KUB X-ray: Kidney urinary bladder X-ray; LUTS: Lower Urinary Tract Symptoms; US: Ultrasonography; TRUS: Transrectal Ultrasonography; TURP: Transurethral prostatectomy

Not long ago, Carneiro et al. mentioned a 2 cm JC incidental detection in an elder male during a preoperative workup for colorectal cancer [17]. Intriguingly, Roose et al. commented on the patient’s preference against an active treatment of JC despite the radiologic findings of prostate enlargement and bladder diverticulum [3]. In another interesting article, Brogna et al. cited the incidental detection of JC in an 84-year-old male with nonspecific abdominal pain [9].

Contrary to popular belief, JC can also be found in the kidney, with such an exceedingly rare case being described in a 63-year-old woman presenting with intermittent visible hematuria of 4-month duration. The stone, measuring 2.4×2.3 cm in size, was successfully fragmented with laser retrograde intrarenal surgery [18]. Unfortunately, literature on upper urinary tract JC is deemed scarce and more reports should ensue [18,19].

Ultrasound imaging is highly endorsed by the European Association of Urology (EAU) as the first-line diagnostic modality for bladder lithiasis [20]. Indeed, any clinical doubt justifies investigation with noncontrast CT, while KUB X-ray aids in treatment planning and follow-up in patients with confirmed vesical stones. Moreover, cystoscopy can be both diagnostic and therapeutic [7,10,20].

The optimal management of bladder lithiasis remains a long-running dispute as diverse therapeutic strategies virtually exist [7,10]. Equipment availability, operative costs, individual characteristics, surgical experience, and stone parameters should be carefully weighed before any potential intervention [10]. Available treatment options encompass conservative treatment, extracorporeal shock wave lithotripsy, open suprapubic cystolithotomy, and modern endourological procedures, either transurethral or percutaneous [7,10,20]. Overall, transurethral cystolithotripsy excels in the length of hospital stay and rates of major complications compared to open surgery. The latter entails a viable solution in cases with considerable stone burden, albeit with the downsides of higher morbidity and longer duration of hospital stay [20]. In recent years, the endoscopic route attracted significant interest owing to the safe, efficient, and reliable clinical application of Holmium:YAG laser cystolithotripsy. Mechanical, ultrasound, electrohydraulic, Swiss Lithoclast, and Neodymium:YAG laser procedures have been all tested for transurethral stone disintegration as well [10].

Modern surgical advancements have introduced minimally invasive approaches for vesical calculi removal, thus expanding the therapeutic scope [6,21]. Although being the gold standard for various urological pathologies, laparoscopic surgery has been rarely used for bladder lithiasis. Fortunately, improvements in conventional laparoscopy have broadened this concept to a novel singlesite technique conferring minimal invasiveness and morbidity [6,22,23]. As such, laparo-endoscopic single-site surgery (LESS) gained traction as a promising alternative to traditional laparoscopy, and, in 2019, Zhang et al. presented their compelling experience with robot-assisted LESS in a 49-year-old male with multiple urinary tract calculi. The authors successfully removed three bladder stones along with three renal and one ureteral calculi through a single-site port without major complications [6]. Moreover, an innovative, safe, and efficient transvesical LESS (T-LESS) for intact medium-size or multiple stone removal was illustrated by Roslan et al. with the aid of a single-port device (Tri-Port+, Olympus, Hamburg, Germany) [22]. As the number of urologists embracing LESS is expected to grow shortly, further studies assessing the method’s reproducibility are certainly warranted.

Lastly, the long-standing issue of whether prostate surgery should be an adjunctive procedure to stone surgical removal continues to demand answers [10]. Currently, EAU Guidelines strongly recommend that once indicated, surgery for bladder outflow obstruction should be concurrently performed with bladder stone removal [20]. Nevertheless, several groups have questioned this approach pointing towards medical management of BPH as an adjunct to surgical stone removal [10]. In our case, stone’s morphology and BPH refractory to combination medical treatment have prompted us to proceed with transvesical prostatectomy and simultaneous stone clearance.

## Conclusion

Βladder lithiasis constitutes a medical problem afflicting 5% of adult males in the Western world. Clinicians should be aware of Jackstone urolithiasis, that once detected, necessitates a patient-tailored approach. Diverse therapeutic options are readily available while eliminating the underlying cause remains the cornerstone of treatment. Our case adds to the growing body of evidence, further providing valuable insights into a rare urological entity. The authors hope that this case report will lead to prompt diagnosis and proper treatment in any such case encountered.
